# Organophosphate Esters in Indoor Environment and Metabolites in Human Urine Collected from a Shanghai University

**DOI:** 10.3390/ijerph18179212

**Published:** 2021-08-31

**Authors:** Yujie Wang, Ming Yang, Fushun Wang, Xueping Chen, Minghong Wu, Jing Ma

**Affiliations:** School of Environmental and Chemical Engineering, Shanghai University, Shanghai 200444, China; wyj434880134@shu.edu.cn (Y.W.); mingyang@shu.edu.cn (M.Y.); fswang@shu.edu.cn (F.W.); xpchen@shu.edu.cn (X.C.); mhwu@shu.edu.cn (M.W.)

**Keywords:** indoor exposure, organophosphate esters, organophosphate ester metabolites, urine, external exposure, internal exposure

## Abstract

In China, organophosphate esters (OPEs) are widely used in indoor environments. However, there is little information regarding the internal and external exposure of university students to OPEs. Therefore, in this study, nine OPEs and eight OPE metabolites (mOPEs) were measured in indoor dust and atmospheric PM_2.5_ samples from a university campus in Shanghai, as well as in urine samples collected from the university students. The total concentration of OPEs in the indoor dust in female dormitories (1420 ng/g) was approximately twice that in male dormitories (645 ng/g). In terms of indoor PM_2.5_, the highest OPE concentration was found in meeting rooms (105 ng/m^3^, on average), followed by chemical laboratories (51.2 ng/m^3^), dormitories (44.9 ng/m^3^), and offices (34.9 ng/m^3^). The total concentrations of the eight mOPEs ranged from 279 pg/mL to 14,000 pg/mL, with a geometric mean value of 1590 pg/mL. The estimated daily intake values based on the indoor dust and PM_2.5_ OPE samples (external exposure) were 1–2 orders of magnitude lower than that deduced from the concentration of urinary mOPEs (internal exposure), indicating that dermal contact, dust ingestion, and inhalation do not contribute significantly to OPE exposure in the general population. Moreover, additional exposure routes lead to the accumulation of OPEs in the human body.

## 1. Introduction

The indoor environment has the most direct impact on human health and socioeconomic development [1]. Poor indoor environments increase the burden on human immune systems, leading to asthma, leukemia, immune infertility, lung cancer, and other diseases [2,3]. With increasing industrial development in China, new building materials, decoration materials, and consumer goods are produced and used in large quantities, along with decreased ventilation in energy-efficient buildings [4]. These developments have led to the emergence of organic compounds, such as flame retardants, plasticizers, surface antifouling agents, and surfactants, which were rarely found in Chinese indoor environments decades ago but are now important “modern” indoor exposure factors.

As substitutes of brominated flame retardants, organophosphate esters (OPEs) are widely used as flame retardants, plasticizers, stabilizers, and defoamers for various consumer and industrial products because of their excellent flame retardant performance and flexibility [5]. According to the Chinese Flame Retardant Industry Report (2014–2016), the market share of OPEs produced in China accounts for 30% of the global flame retardant usage (approximately 620,000 tons) [6]. As OPEs are not chemically bound to the product, they are likely to be released into the ambient environment from the production and use of these materials via volatilization, abrasion, diffusion, and leaching processes [7]. OPEs have been widely detected in various environmental matrices and organisms, including the atmosphere [8], water and sediments [9,10,11], indoor dust [12,13], soil [14], and fish [15]. OPEs in the environment can enter human bodies through dermal contact, dust ingestion, inhalation, and dietary intake, and subsequently undergo accumulation. Considering human breast milk as an example, the mean OPE content in breast milk (3.61 ng/mL) [16] was much higher than that of perfluoroalkyl substances (0.197 ng/mL) [17] and was close to that of polybrominated diphenyl ethers (3.81 ng/g, converted from the average lipid content) [18]. Recent studies have found that OPEs have various negative effects, including neurotoxicity, reproductive toxicity, carcinogenicity, endocrine disruption, and genetic toxicity, and their environmental hazard potentials are gradually being confirmed [19,20]. Tri(1,3-dichloro-2-propyl) phosphate (TDCIPP), tris(1-chloro-2-propyl) phosphate (TCIPP), and triphenyl phosphate (TPHP) have mutagenic and carcinogenic effects, which alter human thyroid hormone levels [21,22] and lead to endocrine disruption [23]. As a neurotoxin, TDCIPP [24] can also lead to asthma. Meanwhile, TPHP has been proven to present contact allergy effects and adverse effects on fertility, while TDCIPP has been related to changes in male hormone levels and a decrease in semen quality [25]. As these chemicals are continuously released from OPE-containing materials in enclosed environments, particles are often preferentially adsorbed on indoor dust and air with potential adverse health effects via indoor exposure. Therefore, indoor exposure to OPEs is attracting increasing research attention [26].

With modernization and urbanization, China’s indoor environment has undergone tremendous changes; moreover, China presents unique population and exposure characteristics. To date, research on indoor exposure to OPEs has mainly focused on the family environment [7,27,28]. However, the information available regarding the indoor operating environment in universities is limited, which includes offices where projectors, computers, printers, and electronic equipment are widely used; chemical analysis laboratories; or crowded university dormitories. Therefore, we need to obtain more data on the characteristics of OPE pollution in different indoor environments to assess the health risks for full-time university students exposed to OPEs. In general, OPEs are easily metabolized into their respective diesters in the human body [29] and produce various phase II conjugate metabolites [30,31,32,33]. The existence of OPE metabolites in human urine, which is relatively easy to collect via biological monitoring, could provide information regarding the in vivo exposure dose.

Environmental pollution likely enters the human body through different ways after contact with human body; this is considered as external exposure. On the other hand, initial chemical dosage absorbed and distributed throughout the body via systemic circulation is considered as internal exposure. In this study, indoor dust and fine particulate matter (PM_2.5_) samples were collected from a dormitory, office, meeting room, and chemical laboratory of a university in Shanghai. Urine samples were collected from university students aged 22–30 years. Nine OPEs and eight mOPEs were selected as target compounds. The purpose of this study was to (a) determine the concentration, profile, and lifetime carcinogenic risk of OPEs in different indoor environments of a university and (b) investigate the levels of mOPEs in the urine samples of university students, infer the actual exposure level according to the internal exposure dose of mOPEs, and compare this value with the estimated external exposure level.

## 2. Materials and Methods

### 2.1. Sample Collection and Chemicals

Sampling was conducted from December 2017 to July 2018 at Shanghai University, which is located in the suburban area of Shanghai, China. PM_2.5_ samples (*n* = 37 in total) were randomly selected from offices (*n* = 12), chemical analysis laboratories (*n* = 9), dormitories (*n* = 12), and meeting rooms (*n* = 4). Quartz filters (QMA, Whatman, Boston, MA, USA) with a medium flow air particulate matter sampler equipped with a cutting head for 2.5 μm particles (TH-150, Tianhong, Wuhan, PRC) were applied at a sampling flow of 0.1 m^3^/min for 8 h. Indoor dust samples were collected from female (*n* = 48) and male dormitories (*n* = 47). A household vacuum cleaner was used to collect indoor dust from apartment dormitories (Figure S1 in the Supporting Information). All the collected quartz filters and screened dust samples were stored in a freezer at −29 °C for pretreatment. Morning urine samples (*n* = 60) were collected from female (*n* = 24) and male university students (*n* = 36) recruited from Shanghai University. The study protocol was reviewed and approved by the Ethics Committee of Shanghai Zhabei District Shibei Hospital. Each volunteer was asked to fill out a questionnaire, including information such as age, sex, smoking status, allergy history, and use of electronic devices. The collected urine samples were immediately transferred to a laboratory freezer maintained at −29 °C.

Nine triester OPEs and eight diester mOPEs were analyzed: triethyl phosphate (TEP), tri-n-propyl phosphate (TPP), tris(2-butoxyethyl) phosphate (TBOEP), tris(2-ethylhexyl) phosphate (TEHP), TCIPP, TDCIPP, TPHP, tris(methylphenyl) phosphate (TMPP), tris(2-chloroethyl) phosphate (TCEP), diphenyl phosphate (DPHP), di(methylphenyl) phosphate (DMPP), diethyl phosphate (DEP), bis(2-ethylhexyl) phosphate (BEHP), bis(2-butoxyethyl) hydrogen phosphate (BBOEP), bis(2-chloroethyl) phosphate (BCEP), bis-(1-chloro-2-propyl) phosphate (BCIPP), and bis(1,3-dichloro-2-propyl) phosphate (BDCIPP). More details are provided in the Supporting Information (Table S1).

### 2.2. Chemical Analysis

OPEs and mOPEs were extracted from indoor dust/PM_2.5_ and human urine samples, respectively. Nine OPEs were analyzed using a gas chromatography-mass spectrometer (GC/MS, 7890A/5975C, Agilent, CA, USA). Eight mOPEs were analyzed using an Agilent 1260 liquid chromatograph coupled with an Agilent 6460 triple quadrupole mass spectrometer (Agilent, Palo Alto, CA, USA). Details regarding sample preparation and instrumental analysis are provided in the Supporting Information.

### 2.3. Quality Assurance and Quality Control

Every 10 samples were equipped with a procedural blank to monitor contamination and environmental interference during the experimental operation. The limit of quantification (LOQ) was set to three times the standard deviation of the blank samples. The recoveries of the two internal standards of OPEs were as follows: 82% ± 19% for d_15_-TPHP, and 89% ± 20% for triamyl phosphate. The recoveries of deuterated internal standards for mOPEs were as follows: 90% ± 18% for d_10_-DPHP, 93% ± 16% for d_10_-BDCIPP, and 91% ± 20% for d_8_-BBOEP. The LOQ values of the OPEs in the indoor dust and PM_2.5_ were 0.24–21.5 ng/g and 10.0–1340 pg/m^3^, respectively. Meanwhile, the LOQ of the mOPEs in the urine samples ranged from 0.84 pg/mL to 21.9 pg/mL.

### 2.4. Statistical Analysis

Considering biological diversity, the specific gravity (SG) of the urine was used to correct the concentration of the target substance in the urine. The concentration of the object below the detection limit was set to 0. Further, 1/2 LOQ was used as the substitute for concentrations below the LOQ. Positive matrix factorization (PMF) was used to estimate the source information of the target analytes in different media. Statistical analyses were performed using Origin 8.0 (OriginLab Corporation, Northampton, MA, USA) and SPSS (version 19.0, SPSS Inc., Chicago, IL, USA).

## 3. Results and Discussion

### 3.1. Concentration Distribution of OPEs in Indoor Dust and Atmospheric PM_2.5_

**Figure 1:**
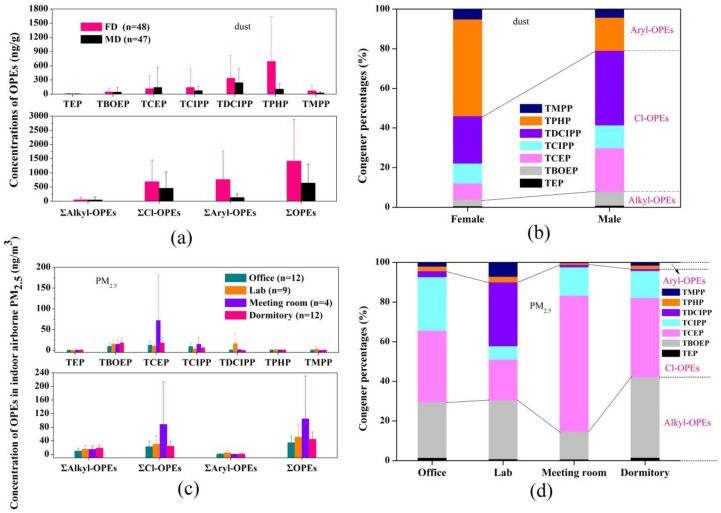
(**a**) Concentrations of OPEs in indoor dust samples from male dormitories (MD) and female dormitories (FD) (ng/g); (**b**) Composition of OPEs in indoor dust samples from male dormitories (MD) and female dormitories (FD) (%); (**c**) Concentrations of OPEs in indoor atmospheric PM_2.5_ samples from different functional areas (ng/m^3^); (**d**) The composition of OPEs in indoor atmospheric PM_2.5_ samples from different functional areas (%).

Seven of the nine targeted OPE compounds were found in both the indoor dust and PM_2.5_ at detection rates ranging from 78–99% and 97–100%, respectively, indicating that OPEs are widespread in indoor campus environments. Therefore, ΣOPEs are considered as the sum of concentrations of these seven individual compounds (TEP, TCEP, TCIPP, TDCIPP, TPHP, TBOEP, and TMPP). The concentrations of ΣOPEs in indoor dust and PM_2.5_ are shown in Figure 1, and the distribution of the OPE data is listed in Table S2. The concentration range of ΣOPEs was 0.39–6480 ng/g (mean value: 1040 ng/g) in the indoor dust samples. Specifically, TPHP (39.0%) was the predominant compound, followed by TDCIPP (28.1%). A similar profile was observed for the indoor dust of a Canadian e-waste dismantling facility [34]; e-waste recycling regions in Guangdong, China [35]; and several microenvironmental floors in Beijing, China [36]. The concentration range of ΣOPEs was 0.015–287 ng/m^3^ (mean value: 50 ng/m^3^) in the indoor PM_2.5_, wherein TCEP (40.8%) was the predominant compound, followed by TBOEP (29.0%). A similar pattern was observed in the indoor PM_2.5_ of classrooms in Norway [37]. Cl-OPEs presented the highest contribution to indoor dust (51.0%) and PM_2.5_ samples (65.2%). The ΣOPE concentration measured in the female dormitory (1420 ng/g) was approximately twice that measured in the male dormitory (645 ng/g). Aryl-OPEs and Cl-OPEs presented higher contributions in female dormitories, whereas Cl-OPEs were dominant in male dormitories (Figure 1). TPHP (48.9%), TDCIPP (23.8%), and TCIPP (10.0%) presented the highest contributions to the dust in the female dormitories, while TDCIPP (37.7%), TCEP (22.1%), and TCIPP (11.5%) were dominant in the male dormitories. TPHP has been detected in nail polish, and DPHP was measured in urine samples from female participants who applied nail polish [38]. This indicates that the different lifestyles of males and females can affect their exposure to the indoor environment. The concentrations of OPEs in indoor dust have been reported in many countries (Table S3). In general, the concentrations of OPEs in this study were similar to those reported for Kuwait, New Zealand, Germany, and Saudi Arabia [39,40,41,42], which were one order of magnitude lower than those reported in developed countries, such as the United Kingdom, Sweden, and Japan [43,44,45], and nearly one order of magnitude higher than those reported in Pakistan, Egypt, and Nepal [13,39,46].

Regarding indoor PM_2.5_, the highest concentration of ΣOPEs was found in meeting rooms (105 ng/m^3^, on average), followed by chemical laboratories (51.2 ng/m^3^), dormitories (44.9 ng/m^3^), and offices (34.9 ng/m^3^). Cl-OPEs were the dominant OPE compounds in indoor air PM_2.5_, followed by alkyl-OPEs. The OPE profiles were similar among offices (TCEP, 36.2%; TBOEP, 28.0%; and TCIPP, 27.0%), meeting rooms (TCEP, 68.6%; TCIPP, 14.3%; and TBOEP, 14.0%), and dormitories (TBOEP, 40.7%; TCEP, 39.9%; and TCIPP, 13.6%), but different from those in laboratories (TDCIPP, 32.2%; TBOEP, 29.8%; and TCEP, 20.6%). Previous studies have mostly focused on indoor air in homes and workplaces (e.g., offices, laboratories, and shops); for comparison, these values are listed in Table S4. In general, the OPE concentration (mean value: 50 ng/m^3^, range: 0.015–287 ng/m^3^) in this study was similar to the levels of ΣOPEs reported in Switzerland (3.90–270 ng/m^3^) [47] and Spain (1.59–202 ng/m^3^) [48], but was several orders of magnitude lower than those reported in developed countries, such as Sweden (101–1900 ng/m^3^) [49], the United States (2220–1,040,000 ng/m^3^) [50], and Vietnam (540–13,000 ng/m^3^) [51].

### 3.2. Source Analysis for Indoor Dust and Atmospheric PM_2.5_

**Figure 2:**
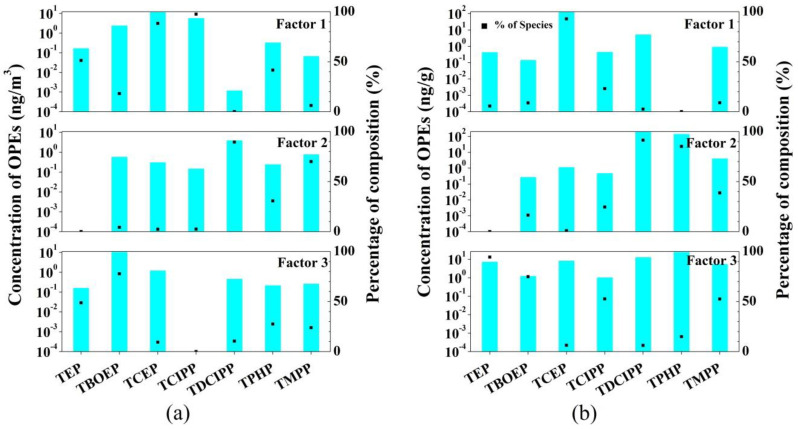
(**a**) The source distribution of OPEs in indoor atmospheric PM_2.5_ (left column: contribution of various factors to the concentration of OPEs (ng/m^3^); right column: impact ratio of various factors on OPEs (%)); (**b**) The source distribution of OPEs in indoor dust (left column: contribution of various factors to the concentration of OPEs (ng/g); right column: impact ratio of various factors on OPEs (%)).

The OPE compound concentrations were determined using the United States Environmental Protection Agency (USEPA) PMF 5.0 model to evaluate the contribution of OPE sources to the indoor environments of the university (additional details in the Supporting Information (Table S9)). According to the PMF software analysis, three key factors were extracted from the indoor dust and PM_2.5_ in the study area. For both indoor dust and PM_2.5_ (Figure 2), the markers of factor 1 were mainly TCIPP and TCEP. Specifically, large amounts of TCEP are used in buildings, and may remain active sources for several years [52]. Meanwhile, TCIPP is a common substitute for pentabromodiphenyl ether in polyurethane foam [19]. Thus, it was preliminarily speculated that factor 1 was indicative of the release from building materials and furniture. For these OPEs, inhalation is expected to be a more dominant intake pathway than dust ingestion and dermal contact [53]. Factor 2 was characterized by high loadings of TDCIPP, TPHP, and TMPP. TDCIPP is commonly used as an additive in polyurethane foam padding used in furniture, children’s foam products, and automobile upholstery [54,55]. Frequent use of electronics was associated with higher TDCIPP hand wipe levels [56]. TPHP is one of the most effective flame retardants for many polymers and can be used in hydraulic fluids [52] and polyvinyl chloride (PVC) [57]. Zheng et al. [35] found that TPHP was the main OPE in the indoor dust at e-waste recycling stations in South China. TMPP can also be used in hydraulic fluids [52] and PVC [57]; it has also been reported to be the main organophosphate flame retardant in e-waste disposal sites in southern China [58]. Therefore, factor 2 was likely attributed to the use of electronic equipment. Factor 3 was heavily loaded with TBOEP and TEP. Alkyl-OPEs are mainly used as plasticizers in unsaturated polyester resins, cellulose acetate, PVC, synthetic rubber, and other materials [59]. In addition, they can also be used as defoaming agents in coatings, hydraulic oils, and floor waxes [60]. TBOEP has been reportedly used as an additive in floor polishing in school indoor environments [37]. Therefore, factor 3 was speculated to be indicative of the use of decoration materials or other consumer goods.

### 3.3. Concentration Distribution of mOPEs in Human Urine Samples

**Figure 3:**
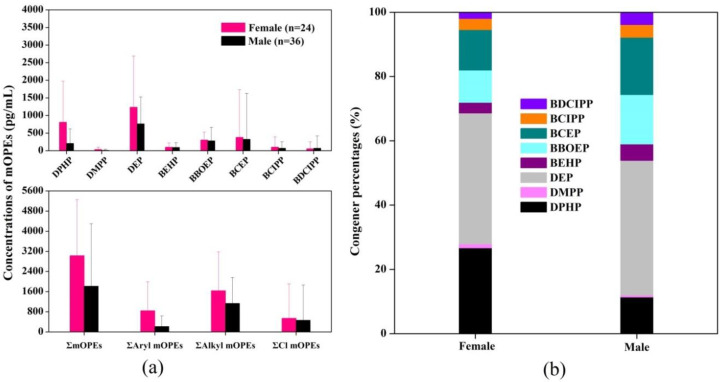
(**a**) Concentrations of mOPEs in male and female urine samples (pg/mL); (**b**) Composition of mOPEs in male and female urine samples (%).

The SG-adjusted geometric mean (GM) concentrations and profiles of eight mOPEs in the collected urine samples are illustrated in Figure 3, and the distribution of the mOPE data is listed in Table S5. Aryl-mOPEs (DPHP, 78.3%) and alkyl-mOPEs (DEP, 91.7%; BBOEP, 78.3%; and BEHP, 75.0%) presented high detection frequencies in all urine samples. Cl-mOPEs were observed with detection frequencies of less than 50%, for which similar patterns were reported for the detection frequencies of urinary mOPEs in a general exposed population in China [61]. The total concentrations of the eight mOPEs (ΣmOPEs) ranged from 279 pg/mL to 14,000 pg/mL, with a GM value of 1590 pg/mL. The ΣmOPE concentrations in the female urine samples were higher than those in the male urine samples, but the difference was not significant (*p* > 0.05). Alkyl-mOPEs were the predominant mOPEs in both male and female urine samples. The proportion of dominant mOPE monomers in the female urine samples was as follows: DEP (40.8%) > DPHP (26.7%) > BCEP (12.6%). In contrast, in the male urine samples, the proportion of dominant mOPE monomers was DEP (41.9%) > BCEP (17.8%) > BBOEP (15.4%). It has been reported that there are significant differences between males and females in terms of exposure, toxicokinetics, and reactions to chemicals [62,63]. The results in this study show that, except for DPHP, gender differences had no significant effect on the distribution of mOPEs in urine samples. However, the small sample size analyzed in these studies may be subject to certain biases.

The main mOPE monomers reported in China and globally were BDCIPP and DPHP, which were 2–3 orders of magnitude higher than the concentration levels in this study, indicating that the internal exposure degrees of TDCIPP and TPHP in this study area were relatively low (Table S6). This might be attributed to the different indoor uses of OPEs; for example, the United States has a high demand for flame retardant interior furniture [31,64]. Although organic toxic pollutants in the environment may form pollution sinks in the indoor environment, pollutants such as OPEs physically added to the products may function as a pollution source. We also compared the urinary mOPE concentrations with the concentrations of their corresponding parent OPEs in indoor environments on campus and found no significant correlation (Tables S7 and S8). This may be because indoor atmospheric particulate matter or dust concentrations only reflect the exposure pathways of dermal contact and respiratory intake, whereas, for the general population, dietary intake is the predominant pathway. In addition, there are differences in the metabolic mechanism of each OPE upon entry into the human body [65].

### 3.4. Risk Assessment of OPEs

Details regarding the estimated daily intake (EDI), hazard index (HI), and carcinogenic risk (CR) of OPEs via air and dust exposure are provided in the Supporting Information (Tables S10 and S11). The average and high exposure EDI*_total_* values of students with seven OPEs, as estimated by indoor air intake, dust intake, and skin absorption, were 5.10 ng/kg bw/d and 14.0 ng/kg bw/d, respectively. The daily exposure dose of OPEs calculated by indoor air intake and dust intake was similar to the EDI value of the drinking water intake of OPEs reported in New York [66], and approximately 10 times lower than the OPE EDI values of indoor air intake and dust intake in the United States [50]. The EDI*_inhalation_* value of OPEs was higher than that of the indoor air PM_2.5_ of subway station [48], while EDI*_ingestion_* was lower than that in the indoor dust in Guangzhou [7]. In general, the EDIs of OPEs in indoor air and dust in this study were in the low-middle level. The HI value of the OPE monomers with non-potential carcinogenic risk was less than 1, and the CR value of the OPE monomers with a potential carcinogenic risk was less than 1 × 10^−6^, which indicates that there was no potential health risk when exposed to indoor air PM_2.5_ and indoor dust in this university.

mOPEs in urine are non-invasive biomarkers that can be used to identify and quantify human exposure to OPEs. They provide comprehensive information on system load, including all types of sources and exposure routes (such as inhalation, dermal contact, and oral ingestion), and can be used to quantify personal exposure. Biotransformation can be an important determinant of the toxicological effects and bioaccumulation of xenobiotics [32]. Despite the limitations of the OPE kinetic or metabolic data in terms of the human body [67], metabolism studies of five mOPEs (TCEP, TCIPP, TDCIPP, TPHP, and TBOEP) in human liver microsomes (HLMs) and S9 fractions provided some evidence of their bioavailability and toxicity in humans [32]. Herein, the individual internal exposure OPE doses according to the concentration of related mOPEs in the urine samples were estimated (Table 1). For EDI_HLM_, the exposure level of donors revealed that the concentration of TCEP was the highest (103 ng/kg bw/day), followed by TPHP (52.5 ng/kg bw/day), TBOEP (8.07 ng/kg bw/day), TCIPP (4.97 ng/kg bw/day), and TDCIPP (3.57 ng/kg bw/day). For EDI_S9_, this order was TPHP (118 ng/kg bw/day), TCEP (55.8 ng/kg bw/day), TBOEP (40.9 ng/kg bw/day), TCIPP (5.86 ng/kg bw/day), and TDCIPP (2.42 ng/kg bw/day). All the estimated HI levels were less than 1, suggesting that there was no significant risk of exposure to OPEs in this study.

In addition, it was found that the OPE EDI values estimated by exposure to indoor dust and PM_2.5_ (external exposure) were 1–2 orders of magnitude lower than that deduced from the concentration of urinary mOPEs (internal exposure), indicating that dermal contact, dust ingestion, and inhalation do not contribute significantly to OPE exposure in the general population. Studies have found that dietary intake is another important means of human exposure to OPEs [68]. TCEP has been detected in eggs from southern China at a detection rate of 100% [69]. A comprehensive investigation of OPE concentrations in Chinese food indicated that TCEP (mean value: 0.74–29.8 ng/g) was the most important OPE in various food categories, including rice, cereals, vegetables, meat, and fruits [70]. In China, rice intake is considered to be the main means of exposure to OPEs in foods, as it presents the highest contribution to total intake, accounting for approximately 60% [71]. The highest levels of OPEs have been reported in cereals, which may be the main path of dietary intake for the Chinese population [72].

## 4. Conclusions

The OPE concentrations and EDIs in the indoor dust and atmospheric PM_2.5_ samples obtained in this study were relatively low, as compared with global levels. Specifically, the concentration level of mOPEs was 2–3 orders of magnitude lower than that reported in domestic and foreign studies. Three key contributing factors were extracted using the PMF model of the OPE concentrations in the indoor dust and PM_2.5_ samples, revealing that building materials and furniture, electronic equipment, and the use of decoration materials and other consumer goods were the main contributing factors. The OPE EDI values estimated using the OPE indoor dust and PM_2.5_ samples were 1–2 orders of magnitude lower than that deduced by urinary mOPEs. These findings can act as a foundation for the establishment of a comprehensive evaluation mechanism for indoor and outdoor environments in the future. Upon comparing internal and external exposure, it was found that dermal contact, dust ingestion, and inhalation did not contribute significantly to OPE exposure in the general population. Instead, dietary intake may be the main exposure pathway contributing to the health risks of the general population. Full-time college students typically reside and study on campus for several years, which presents a good research object for studying the health effects of indoor exposure. In the future, extensive research should be carried out, including on dietary exposure, in order to obtain more accurate data on the exposure to OPEs in the general population.

## Figures and Tables

**Table 1:** EDIs (ng/kg bw/d), HI, and CR from the exposure of OPEs.

Analytes		TEP	TBOEP	TCEP	TCIPP	TDCIPP	TPHP	TMPP	ΣOPEs	Alkyl-OPEs	Cl-OPEs	Aryl-OPEs
RfD		1.30 × 10^5^	1.50 × 10^4^	7.00 × 10^3^	1.00 × 10^4^	2.00 × 10^4^	7.00 × 10^4^	1.30 × 10^4^				
SFO				2.00 × 10^−8^		3.10 × 10^−8^						
**EDIs (ng/kg bw/d), HI and CR from the exposure of OPEs in indoor dust and air**												
EDI*_ingestion_*	Average	1.33 × 10^−3^	5.24 × 10^−3^	6.45 × 10^−3^	5.30 × 10^−3^	3.36 × 10^−2^	3.29 × 10^−2^	5.88 × 10^−3^	1.61 × 10^−1^	7.26 × 10^−3^	6.95 × 10^−2^	3.76 × 10^−2^
	High	2.95 × 10^−3^	1.69 × 10^−2^	4.81 × 10^−2^	4.04 × 10^−2^	1.08 × 10^−1^	1.50 × 10^−1^	1.85 × 10^−2^	3.85 × 10^−1^	1.99 × 10^−2^	1.97 × 10^−1^	1.69 × 10^−1^
EDI*_dermal adsorption_*	Average	1.41 × 10^−3^	5.55 × 10^−3^	6.84 × 10^−3^	5.62 × 10^−3^	3.56 × 10^−2^	3.49 × 10^−2^	6.24 × 10^−3^	1.71 × 10^−1^	7.70 × 10^−3^	7.37 × 10^−2^	3.99 × 10^−2^
	High	3.79 × 10^−3^	2.17 × 10^−2^	6.18 × 10^−2^	5.18 × 10^−2^	1.39 × 10^−1^	1.93 × 10^−1^	2.37 × 10^−2^	4.95 × 10^−1^	2.55 × 10^−2^	2.53 × 10^−1^	2.17 × 10^−1^
EDI*_inhalation_*	Average	5.25 × 10^−2^	1.55	1.47	7.22 × 10^−1^	1.04 × 10^−1^	8.16 × 10^−2^	4.41 × 10^−2^	4.77	1.68	3.15	1.46 × 10^−1^
	High	1.54 × 10^−1^	3.79	5.34	1.98	1.22	2.56 × 10^−1^	3.54 × 10^−1^	1.31 × 10^1^	3.95	8.53	6.10 × 10^−1^
EDI*_total_*	Average	5.52 × 10^−2^	1.56	1.48	7.33 × 10^−1^	1.73 × 10^−1^	1.49 × 10^−1^	5.62 × 10^−2^	5.10	1.69	3.30	2.24 × 10^−1^
	High	1.60 × 10^−1^	3.83	5.45	2.07	1.46	5.99 × 10^−1^	3.97 × 10^−1^	1.40 × 10^1^	3.99	8.98	9.95 × 10^−1^
HI	Average	4.25 × 10^−7^	1.04 × 10^−4^	2.11 × 10^−4^	7.33 × 10^−5^	8.66 × 10^−6^	2.13 × 10^−6^	4.33 × 10^−6^				
	High	1.23 × 10^−6^	2.55 × 10^−4^	7.78 × 10^−4^	2.07 × 10^−4^	7.32 × 10^−5^	8.55 × 10^−6^	3.05 × 10^−5^				
CR	Average			2.96 × 10^−8^		5.37 × 10^−9^						
	High			1.09 × 10^−7^		4.54 × 10^−8^						
**EDIs (ng/kg bw/day) and HI calculated under various assumptions of urine excretion of OPEs in different sex groups**												
Daily intakes estimated based on F_ue_ values of OPEs in human liver microsomes system (EDI_HLM_)												
EDI_total_	Male		9.22	118	5.68	4.08	60		197			
	Female		6.92	88.8	4.26	3.06	45		148			
HI	Male		6.15 × 10^−4^	1.69 × 10^−2^	5.68 × 10^−4^	2.04 × 10^−4^	8.58 × 10^−4^		1.92 × 10^−2^			
	Female		4.61 × 10^−4^	1.27 × 10^−2^	4.26 × 10^−4^	1.53 × 10^−4^	6.43 × 10^−4^		1.44 × 10^−2^			
CR	Male			2.36 × 10^−6^		1.26 × 10^−7^						
	Female			1.78 × 10^−6^		9.49 × 10^−8^						
Daily intakes estimated based on F_ue_ values of OPEs in human S9 fraction system (EDI_S9_)												
EDI_total_	Male		46.7	63.8	6.7	2.76	135		255			
	Female		35	47.8	5.02	2.07	101		191			
HI	Male		3.11 × 10^−3^	9.11 × 10^−3^	6.70 × 10^−4^	1.38 × 10^−4^	1.93 × 10^−3^		1.50 × 10^−2^			
	Female		2.33 × 10^−3^	6.83 × 10^−3^	5.02 × 10^−4^	1.03 × 10^−4^	1.45 × 10^−3^		1.12 × 10^−2^			
CR	Male			1.28 × 10^−6^		8.56 × 10^−8^						
	Female			9.56 × 10^−7^		6.42 × 10^−8^						

## Data Availability

All data generated and analyzed during this study are included in this published article and its supplementary information files.

## Supplementary Materials

The following are available online at https://www.mdpi.com/article/10.3390/ijerph18179212/s1, Figure S1: Sample collection from indoor environment, Table S1: Designation and structural formula of OPEs and its metabolites (mOPEs), Table S2: Distribution of OPEs concentrations found in indoor dust (n = 95) and airborne PM_2.5_ (n = 37), Table S3: Concentrations of OPEs in indoor dust worldwide (ng/g), Table S4: Concentrations of OPEs in indoor air (gas and/or PM phase) samples worldwide (ng/m^3^), Table S5: Distribution of SG adjusted OPE metabolite concentrations found in urine (n = 60) from Shanghai (pg/mL), Table S6: SG adjusted geometric mean or median concentrations of urinary mOPEs worldwide (pg/mL), Table S7: Correlation coefficient of mOPEs in human urine and OPEs in indoor dust, Table S8: Correlation coefficients of mOPEs in human urine and OPEs in indoor atmospheric PM_2.5_ samples, Table S9: The parameters of input files in PMF, Table S10: Exposure parameters for children and adults, and Table S11: Parameters used for calculation of total EDI and HI of OPEs in China.
