# Supplementary material for: Organophosphate Esters in Indoor Environment and Metabolites in Human Urine Collected from a Shanghai University
Source: Int J Environ Res Public Health. 2021 Aug 31;18(17):9212. doi: 10.3390/ijerph18179212 (PMC8431728; doi:10.3390/ijerph18179212)
Supplement: Supplementary file 1 [file ijerph-18-09212-s001.zip › ijerph-1305991-supplementary.pdf]

Supporting Information (Supplementary Material)

Chemicals and regents

The standard samples and internal recovery standards of nine organophosphate esters (OPEs) (name, abbreviation, M/Z, and CAS No.), including triethyl phosphate (TEP, 155/99, 78-40-0), tri-n-propyl phosphate (TPP, 183/99, 513-08-6), tris(2-butoxyethyl) phosphate (TBOEP, 299/199, 78-51-3), tris(2-ethylhexyl) phosphate (TEHP, 211/113/99, 78-42-2), tris(1-chloro-2-propyl) phosphate (TCIPP, 277/279, 13674-84-5), tri(1,3-dichloro-2-propyl) phosphate (TDCIPP, 381/379, 13674-87-8), triphenyl phosphate (TPHP, 326/325, 115-86-6), tris(methylphenyl) phosphate (TMPP, 368/367, 1330-78-5), tris(2-chloroethyl) phosphate (TCEP, 249/251, 115-96-8), d_{15}-triphenyl phosphate (d_{15}-TPHP, 341/339/340, 1173020-30-8), and triamyl phosphate (239/169, 2528-36-3), were purchased from Sigma-Aldrich (USA).

Further, standard samples of seven OPE metabolites (mOPEs) and three internal recovery standards (name, abbreviation, molecular weight, quantitative ion/qualitative ion in multiple reaction monitoring mode, and CAS No.), including diphenyl phosphate (DPHP, 250, 249>93.2/249>155, 838-85-7), di(methylphenyl) phosphate (DMPP, 278, 277>107/277>78.8, 843-24-3), bis(2-ethylhexyl) phosphate (BEHP, 322, 321>79.2, 298-07-7), bis(2-butoxyethyl) hydrogen phosphate (BBOEP, 298, 297>79.1/297>197, 14260-97-0), bis(2-chloroethyl) phosphate (BCEP, 223, 221>35.2/223>35.1, 3040-56-0), bis(1-chloro-2-propyl) phosphate (BCIPP, 251, 249>35.2/249>35.2, 789440-10-4), bis(1,3-dichloro-2-propyl) phosphate (BDCIPP, 320, 317>35.2/319>35.2, 72236-72-7), diphenyl phosphate-d_{10} (d_{10}-DPP, 260, 259>98.2/259>159), bis(1,3-dichloro-2-propyl) phosphate ester-d_{10} (d_{10}-BDCIPP, 306, 305>79.0/305>202), and dibutoxyethyl bisphosphate-d_8 (d_8-BBOEP, 329, 327>35.1/329>35.1), were purchased from TRC Inc. (Canada). Meanwhile, a standard sample of diethyl phosphate (DEP, 182, 153>125/153>79.1, 598-02-7) was purchased from the Accustandard (New Haven, USA). The solid phase extraction (SPE) column could be filled with a filter column (disposable consumables, specifications of 3 mL), and the SPE column matching flow valve was purchased from ANPEL Laboratory Technologies (Shanghai) Inc. Further, an amino SPE column (6 mg/3 mL) procured from Phenomenex (USA) was used for the pretreatment analysis of the urine samples.

Sample Preparation

The collected indoor environment samples were stored in a refrigerator and brought to 25 °C; an appropriate amount of the samples was weighed for analysis (dust samples and corresponding blank, 2 g each). The atmospheric particulate matter samples and corresponding blank were obtained from half the quartz membrane, weighed, cut into pieces, and placed in a 50 mL polypropylene centrifuge tube, followed by addition of 40 ng of the internal standard substance. Then, 30 mL of the extraction solution (n-hexane/acetone = 1:1, V:V) was added to the centrifuge tube for carrying out the extraction operation (ultrasound for 30 min, shock for 30 min, and centrifugation for 8 min at 4500 rpm) three times. After each centrifugation operation, the supernatant was collected and transferred to a round-bottomed flask. The extraction solution was concentrated to a small volume (1–2 mL) using a rotary evaporator. The extracted concentrate was then transferred to a rinsed self-filled silica gel purification column (rinsed with 30 mL ethyl acetate and rinsed again with 10 mL n-hexane) and discarded. The column was then eluted with a mixture of 25 mL acetone and dichloromethane (7:3 V:V), and the collected solution was concentrated by rotary evaporation. Finally, the nitrogen was blown through a volume of 200 μL for analysis by a gas chromatography-mass spectrometer (GC/MS).

The thawed urine samples were removed from the refrigerator at 4 °C and brought to 25 °C before the subsequent pretreatment. The creatinine concentration and specific gravity values of the urine samples used were obtained using a U120 Super Analyzer (Aconbiotech, China). Here, 5 mL urine samples (blank is 5 mL ultrapure water) were placed into beakers, followed by addition of 10 ng of three types of deuterium internal

standards. Then, ultrapure water was added to the beaker to dilute the urine sample to a 1:1 ratio and the pH value was checked. If the pH was greater than 7, acetic acid solution (0.1 mol/L) was added to the beaker to achieve a pH between 6 and 7. Next, the SPE column was successively activated with MeOH (3 mL) and ultrapure water (3 mL). The samples were then loaded onto the activated SPE column and the flow rate was controlled at ≤ 1 mL/min). First, the SPE column (3 mL ultrapure water) was washed and dried with a vacuum pump. Then, two elutions were carried out with a 2 mL acetonitrile solution containing 5% pyrrolidine. The collected eluent was concentrated by blowing nitrogen in a water bath (45 °C) until completely dry. The material on the nitrogen blowpipe was dissolved in a 0.5 mL solution (MeOH: ultrapure water = 4:1, V:V). Then, the solution was filtered via a hydrophilic filter membrane (0.22 μm, polytetrafluoroethylene).

Instrumental Analysis

The GC/MS model used for the quantitative analysis of indoor environmental samples was GC/MS 7890A/5975C (Agilent Technologies), and the installed capillary column was DB-5 (30 m × 0.25 mm inner diameter × 0.25 μm). The injection parameters of the instrument in single ion monitoring mode were as follows: automatic injection without shunt (1 L), electron ionization source (70 eV), several specific temperatures (injection port: 280 °C, ion source: 260 °C, four-stage rod: 150 °C, and interface: 280 °C), a certain temperature-raising program (initial temperature: (80 °C for 1 min)→180 °C (heating rate: 10 °C /min; maintained for 1 min)→270 °C (heating rate: 2 °C /min; maintained for 10 min)), and a carrier gas (He) flow rate of 1.82 mL/min.

A liquid chromatograph coupled with an Agilent 6460 triple quadrupole mass spectrometer equipped with a Poroshell 120 EC-C_{18} reversed-phase column (3 mm × 100 mm, 2.7 μm) was used for the quantitative analysis of mOPEs in human urine. The mobile phase consisted of eluent A (MeOH) and eluent B (5 mM NH_4Ac solution). The parameters were set as follows: column temperature: 35 °C; mobile phase: MeOH (eluent A) and NH_4Ac solution (eluent B); sample injection volume: 5 μL; flow rate: 0.4 mL/min; and multiple reaction monitoring mode. The gradient of mobile phase A was 15% (0–0.5 min)→15% increasing to 40% (0.5–3 min) → 40% (3–4.5 min) → 40% rising to 95% (4.5–8 min)→95% (8–18 min)→95% decreasing to 15% (18–20 min)→15% (20–25 min).

Source Analysis

The positive matrix factorization (PMF) model is an improved tool for multivariate factor analysis that is especially suitable for addressing certain limitations of principal component analysis. The PMF incorporates variable uncertainty and identifies factors that explain the source and underlying information regarding the input variable data [1]. PMF can be used for quantitative source contribution analyses. Compared with the chemical mass balance method, it is easier to operate as it does not require the specific chemical composition spectrum information of each pollutant. In recent years, the PMF model has been applied to various media to effectively determine the source and distribution information of target pollutants, such as chlorinated polycyclic aromatic hydrocarbons (PAHs) and PAHs [2,3].

The software used for analysis as well as the data processing involved are summarized in Table S9. The PMF model was used to analyze the dataset of 95 × 7 (95 sites, 7 OPEs) indoor dust and 37 × 7 (37 sites, 7 OPEs) indoor atmospheric $PM_{2.5}$ samples. Through repeated runs of the PMF model (2–7 factors), it was confirmed that the OPE sources in indoor dust and atmospheric $PM_{2.5}$ are affected by three factors. The contribution rates of these factors to the concentration of the seven OPE monomers and the source distribution spectra of the OPE monomers are discussed in this study.

EDI Values of Air and Dust Exposure to OPEs

For two different exposure scenarios, the values of the estimated daily intake $(EDI)_{inhalation}$, $EDI_{ingestion}$, and $EDI_{dermal\ adsorption}$ were calculated for: (a) average exposure,

wherein the OPE concentration is the median concentration, and the indoor air inhalation rate, dust intake rate, or skin adsorption rate are averaged; and (b) high exposure, wherein the average OPE concentration is the 95th percentage point of indoor air inhalation rate, dust intake rate, or skin adsorption rate [4]. Table S10 summarizes the exposure parameters used in this study to assess the exposure risk of OPEs via air and dust.

In this study, the EDIs were estimated using OPEs exposed to air and dust, as follows:

$$EDI_{inhalation} = \frac{C_{air} \times R_{inhalation} \times ED}{BW}$$

$$EDI_{ingestion} = \frac{C_{dust} \times R_{Ingestion} \times ED}{BW}$$

$$EDI_{\text{dermal adsorption}} = \frac{C_{dust} \times SA \times DA \times ADF \times ED}{BW}$$

$$EDI_{total} = EDI_{inhalation} + EDI_{ingestion} + EDI_{dermal\ adsorption}$$

where C_i represents the average concentration of OPEs in air or dust at different sampling points.

The hazard index (HI) was calculated to assess the health risk of exposure to OPEs, which can be defined as the ratio of EDI to reference dose (RfD), using the following equation:

$$\text{HI} = \frac{EDI_{total}}{RfD}$$

where HI is the hazard index of OPEs, EDI_{total} is the estimated daily intake for the human body (ng/kg bw/d), and RfD is the reference dose for OPE exposure (ng/kg bw/d) [4-6]. The HI of each OPE monomer was calculated separately to allow for independent risk assessment of each OPE. If HI < 1, no significant risk of OPE exposure was considered. If HI > 1, there may be risk of exposure to OPEs [7].

The carcinogenic risk (CR) was used to estimate the risk of exposure to carcinogenic or potentially carcinogenic OPEs using the following equation:

$$\text{CR} = EDI_{total} \times SFO$$

Here, SFO is the slope factor (ng/kg bw/d) that indicates the level of CR [4, 6, 8, 9]. For regulatory purposes, the acceptable CR value is considered as between 1×10^{-6} and 1×10^{-4} (with potential carcinogenic risk), wherein a value lower than 1×10^{-6} is considered to indicate no carcinogenic risk, and one higher than 1×10^{-4} is considered to indicate high potential carcinogenic exposure risk [7, 10].

EDIs and Risk Assessment of Exposure to Reversely Derived OPEs in Vivo

Because of the limited kinetic and metabolic data on OPEs in the human body [11], metabolic studies of five mOPEs (TCEP, TCIPP, TDCIPP, TPHP, and TBOEP) in human liver microsomes (HLMs) and S9 components were conducted to provide evidence regarding their bioavailability and toxicity in humans [12]. Therefore, we calculated the EDI data of the F_{ue} of two sets of mOPEs based on the HLM (EDI_{HLM}) and S9 score (EDI_{S9}) systems. The EDIs of the five mOPEs were calculated based on urinary metabolites as follows:

$$\text{EDI} = \frac{C_{mOPEs} \times V_{urine}}{F_{ue} \times BW} \times \frac{MW_p}{MW_m}$$

where C_{mOPEs} is the mOPE concentration in the urine; V_{urine} is the daily urine excretion, which is 1600 mL for males and 1200 mL for females [13]; F_{ue} is the mole fraction of mOPEs in urine relative to its parent compound OPE; BW represents body weight (adult, 80 kg) [13]; and MW_P and MW_m are the molecular weights of OPEs and corresponding mOPEs, respectively. Note that the specific parameters are listed in Table S11.

Finally, HI was calculated to assess the health risk from OPE exposure, which can be

$$\mathrm{HI} = \frac{EDI}{RfD}$$

defined as the ratio of EDI to RfD and is calculated as follows:

Table S1. Designation and structural formula of OPEs and its metabolites (mOPEs).

Full Name (OPEs)	Abbreviation	Full Name (mOPEs)	Abbreviation	Structural Formula (mOPEs)
Triphenyl phosphate	TPHP	Diphenyl phosphate	DPHP	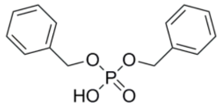
Tris(methylphenyl) phosphate	TMPP	Di(methylphenyl) phosphate	DMPP	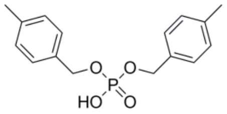
Triethyl phosphate	TEP	Diethylphosphate	DEP	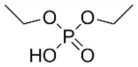
Tris(2-ethylhexyl) phosphate	TEHP	Bis(2-ethylhexyl) phosphate	BEHP	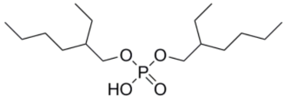
Tris(2-butoxyethyl) phosphate	TBOEP	Bis(2-butoxyethyl) hydrogen phosphate	BBOEP	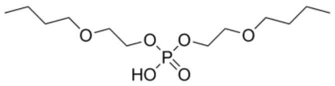
Tris(2-chloroethyl) phosphate	TCEP	Bis(2-chloroethyl) phosphate	BCEP	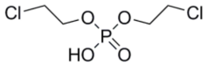
Tris(1-chloro-2-propyl) phosphate	TCIPP	Bis-(1-chloro-2-propyl)phosphate	BCIPP	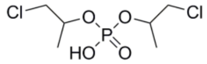
Tri(1,3-dichloro-2-propyl) phosphate	TDCIPP	Bis(1,3-dichloro-2-propyl) phosphate	BDCIPP	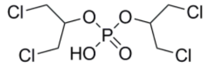
Tri-n-propyl phosphate	TPP			

Table S2. Distribution of OPEs concentrations found in indoor dust (*n* = 95) and airborne $PM_{2.5}$ (*n* = 37).

Analytes	Detection Rate (%)	Maximum	Minimum	Mean	Median	[a] SD	5% Percentile	25% Percentile	75% Percentile	95% Percentile
**Indoor dust (ng/g)**										
TEP	86.3	64.1	< LOQ	7.96	5.36	4.48	< LOQ	2.65	10.4	22.5
TBOEP	77.9	671	< LOQ	45.5	21.2	44.5	< LOQ	3.9	49.9	131

TCEP	90.5	2870	< LOQ	130	26.1	180	< LOQ	6.83	97.4	425
TCIPP	80	2000	< LOQ	109	21.4	142	< LOQ	3.63	110	353
TDCIPP	98.9	1970	< LOQ	291	136	201	1.1	42.5	332	1100
TPHP	94.7	3720	< LOQ	405	133	369	5.49	36.2	266	1880
TMPP	96.8	471	< LOQ	49.8	23.8	42.6	0.58	8.5	47.8	192
ΣOPEs	100	6480	0.39	1040	652	601	108	223	1360	3930
ΣAlkyl-OPEs	100	693	< LOQ	53.5	29.3	46.9	< LOQ	15.3	61.5	147
ΣCl-OPEs	100	3540	0.17	530	281	336	13.1	114	615	1940
ΣAryl-OPEs	100	3960	0.22	455	152	389	19.7	60.8	371	2030
**Indoor airborne $PM_{2.5}$ (ng/m^3)**										
TEP	97.3	2.99	< LOQ	0.58	0.41	0.62	0.12	0.30	0.60	1.52
TBOEP	97.3	29.8	< LOQ	14.4	12.0	9.09	1.19	6.22	18.8	28.5
TCEP	97.3	234	< LOQ	20.3	11.3	38.1	3.00	6.02	19.7	46.9
TCIPP	97.3	35.6	< LOQ	7.51	5.58	7.18	1.13	2.14	9.75	19.1
TDCIPP	97.3	81.0	< LOQ	4.62	0.80	13.9	0.09	0.43	1.78	18.1
TPHP	97.3	3.89	< LOQ	0.97	0.63	0.80	0.36	0.5	1.15	2.48
TMPP	100	17.1	0.01	1.34	0.34	3.10	0.09	0.22	0.95	5.39
ΣOPEs	100	287	0.01	49.7	36.8	48.2	12.6	27.5	53.3	106
ΣAlkyl-OPEs	100	30.3	< LOQ	15.0	13.0	9.02	1.43	8.08	19.1	29.1
ΣCl-OPEs	100	272	< LOQ	32.4	24.4	44.8	6.21	13.3	31.5	68.6
ΣAryl-OPEs	100	20.9	0.01	2.31	1.13	3.77	0.46	0.77	1.89	6.79

[a] SD: standard deviation.

Table S3. Concentrations of OPEs in indoor dust worldwide (ng/g).

	n	TEP	TBOEP	TCEP	TCIPP	TDCIPP	TPHP	TMPP	Ref
Home									
Guangzhou	11		2215 (LOD-23526)	2770 (176-19511)	970 (375-3847)	352 (27-1735)	903 (114-1849)	45.2 (7.24-98.1)	[14]
China	44	13.5 (MDL-2550)	30.2 (0.935-1920)	239 (3.52-3600)	690 (7.41-6730)	120 (MDL-1080)	88.5 (MDL-1310)	8.95 (MDL-182)	[15]

Guangzhou	30		408 (61.6-34240)	417 (95.6-1470)	695 (116-4140)	1360 (455-7560)	608 (133-3550)	258 (110-1400)	[16]
Guangzhou (Urban)	25	120 (20-240)	580 (ND-3050)	5180 (1550-9700)	830 (160-2930)	1260 (ND-9630)	280 (10-800)	840 (ND-7740)	[17]
Guangzhou (Rural)	25	100 (30-410)	350 (30-1760)	2190 (50-9360)	1870 (240-10700)	330 (ND-2700)	1420 (260-5340)	610 (ND-3650)	[17]
Wuhan	53	38.3 (ND-782)	406 (ND-5252)	724 (9.14-3718)	432 (LOD-5474)	190 (ND-1514)	316 (ND-2819)	11.1 (ND-161)	[18]
Beijing	21	58 (ND-350)	8544 (493-41917)	7897 (2231-30847)	2868 (220-13804)	529 (ND-1531)	540 (122-1829)	543 (ND-2327)	[19]
Shanghai	15	200 (20-1400)	5900 (500-27300)	4300 (200-38000)	3800 (600-20600)	700 (200-1700)			[20]
Shanghai	20	80 (LOD-260)	550 (80-2070)	530 (50-3130)	1240 (110-4590)	3510 (420-10190)	50 (LOD-110)		[21]
Nanjing	6			4380 (20-17500)	1270 (80-4930)	6830 (ND-40100)	560 (150-1030)		[22]
Nepal	28			15.7 (0.11-69.1)	61.7 (24.3-805)	22.1 (1.04-1420)	71.3 (9.81-3670)	420 (50.6-4740)	[23]
Philippines, Malate	17			32 (ND-1200)			110 (8.5-440)	13 (ND-140)	[24]
Saudi Arabia, Jeddah	15		580 (ND-2750)	560 (125-1650)	1600 (200-3700)	1770 (150-8700)	310 (65-1200)		[25]
Pakistan, Faisalabad	15	<5 (ND-60)	28.5 (ND-145)	37.5 (ND-175)	18.5 (ND-85)	33 (ND-255)	155 (ND-330)	60 (15-200)	[26]
Kuwait	15	55 (ND-385)	10685 (31-140450)	755 (275-1800)	1955 (120-7065)	530 (60-1555)	1080 (44-6890)	570 (28-6015)	[26]
Japan	10	10 (ND-97)	248521 (3648-1014074)	4065 (871-12091)	2530 (520-8838)	5738 (175-35487)	1369 (233-6655)	2045 (326-8968)	[27]
Egypt, Assiut	20		86 (LOQ-305)	49 (LOQ-132)	53 (LOQ-123)	147 (LOQ-557)	101 (8-289)		[28]
New Zealand	34	10 (ND-72)	4285 (50-27325)	152 (20-7605)	817 (20-7615)	470 (20-16560)	590 (20-7510)	195 (ND-3760)	[29]
Australia	10		20000 (2800-100000)	1500 (150-5800)	14000 (2600-35000)	2800 (900-6500)	720 (310-1600)		[30]

Sweden, Stockholm	10		8500 (600-30000)	7600 (ND-33000)	3100 (700-11000)	12000 (2200-27000)	1600 (100-4200)	1200 (ND-3000)	[31]
Sweden, Stockholm	62	190 (ND-4300)	5700 (ND-107000)	2500 (ND-808000)	4600 (1210-98000)	700 (ND-12000)	2200 (680-38000)	1600 (ND-31000)	[32]
Belgium, Flemish	33		6580 (360-67600)	490 (ND-2650)	4820 (190-73700)	570 (ND-6640)	2020 (40-29800)	440 (ND-5070)	[33]
Spain, Barcelona	5			1790 (134-13200)	2623 (1890-7203)	706 (167-1354)	1102 (580-2633)	278 (103-535)	[34]
Germany	6		730 (LOD-2800)	200 (140-280)	740 (370-960)	<80 (LOD-110)	380 (180-1300)	94 (LOD-240)	[35]
UK, West Mid-lands	32			2200 (LOD-28000)	29000 (3700-100000)	2000 (60-14000)	10000 (490-110000)	2000 (LOD-14000)	[36]
Netherland	8		22000 (4600-159000)	1300 (220-6900)	1300 (480-3800)	280 (70-3200)	820 (680-11000)	110 (LOQ-180)	[37]
Germany	15	310 (LOD-2300)	13000 (2100-99000)	1300 (LOD-5000)	5000 (1700-10000)	850 (LOD-4300)	3600 (480-23000)	430 (LOD-3300)	[38]
USA, Carbondale	17		19500 (6090-713570)	319 (85.6-1820)	5600 (714-51350)	3710 (952-19800)	1580 (655-6430)	272 (10.8-994)	[16]
USA, Washington	20			2660 (ND-25700)	14300 (216-82700)	2580 (570-6380)			[39]
USA, Boston	50				572 (LOD-5490)	1890 (LOD-56090)	7360 (LOD-1798000)		[40]
Romania, Iasi	47	30 (ND-420)	2700 (ND-21000)	800 (LOD-1160)	2500 (LOD-16400)	100 (LOD-460)	1600 (ND-22600)	1000 (ND-5500)	[41]
Brazil	10		15900 (1550-348000)	230 (153-421)	771 (442-2280)	1370 (369-28600)	3900 (542-10700)		[42]
Office									
Guangzhou	22		85.5 (LOD-266)	1158 (105-5199)	2607 (192-8486)	247 (57.5-1038)	744 (120-1715)	77.5 (8.98-260)	[14]
Nanjing	12			3220 (250-15000)	1270 (320-4490)	2410 (390-11700)	1050 (330-2380)		[22]
Beijing	23	170 (LOD-1156)	4576 (1034-14117)	18042 (185-71167)	25117 (308-158014)	1470 (102-5567)	4130 (31-38646)	1372 (LOD-3676)	[19]
Egypt	20		263 (LOD-1244)	61 (LOD-125)	119 (LOD-700)	99 (LOD-490)	94 (11-337)		[28]

Germany	11	820 (250-4300)	45000 (4800-150000)	3700 (930-18000)	52000 (1800-470000)	6300 (800-39000)	3300 (770-9200)	8200 (LOD-48000)	[38]
Germany	10		7000 (2900-13000)	120 (LOD-170)	3000 (180-9400)	150 (LOD-290)	2500 (470-4800)	370 (LOD-1900)	[35]
UK	61			5000 (LOD-160000)	44000 (3600-230000)	2100 (LOD-51000)	8200 (560-50000)		[36]
Sweden	10	100 (ND-300)	87000 (450-960000)	6700 (1300-260000)	19000 (3400-120000)	17000 (3300-91000)	8800 (900-32000)	800 (ND-2900)	[31]
Brazil	5		72800 (23900-1910000)	237 (145-681)	385 (109-69200)	4480 (249-10500)	6420 (1740-47000)		[42]

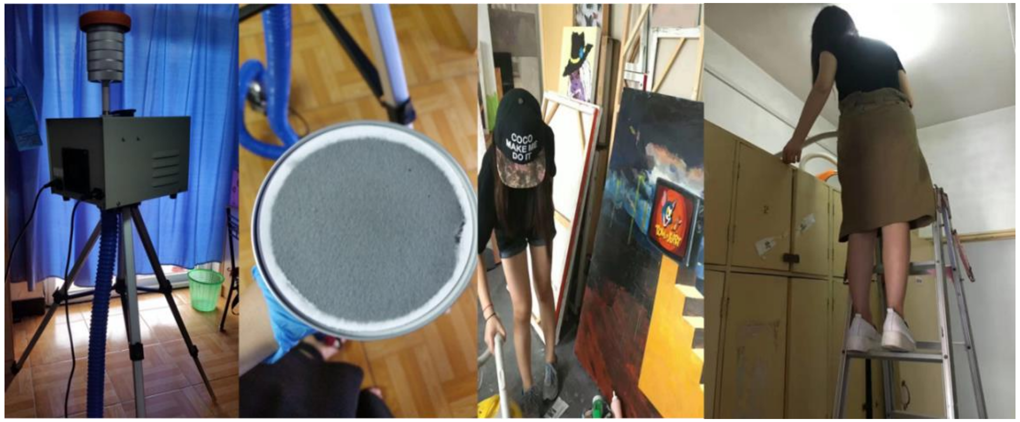

Figure S1. Sample collection from indoor environment.

Table S4. Concentrations of OPEs in indoor air (gas and/or PM phase) samples worldwide (ng/m^3).

Location	Year	Matrix	OPEs (N)	Samples (*n*)	Range	Mean	Median	Ref
Harbin, China	2013–2014	gas	9	25	2.73-18.2	7.43	6.86	[43]
Japan	2013	gas	12	9	<LOQ-1500			[44]
Bihar state, India	2015	gas	8	15	0.09-2.16	0.48	0.35	[45]
New York state, USA	2018	gas	15	54	<LOQ-170	24.9	8.21	[46]
Barcelona, Spain	2013–2016	PM	19	30	1.59-202	39.9	26.2	[47]
New York state, USA	2018	PM	15	54	2220-1040000	77500	36100	[46]

Stockholm, Sweden	2014–2015	PM	10	23	101-1900		340	[48]
Hanoi, Vietnam	2017	PM	10	22	540-13000	2600		[49]
Zurich, Switzerland		gas+PM	8	12	3.9-270			[50]
Oslo and Akershus, Norway	2012	gas+PM	6	54	8.6-467	99	55	[51]

Table S5. Distribution of SG-adjusted OPE metabolite concentrations found in urine (n=60) from Shanghai (pg/mL).

	Aryl-mOPEs			Alkyl-mOPEs				Cl-mOPEs				
	**DPHP**	**DMPP**	**ΣAryl-mOPEs**	**DEP**	**BEHP**	**BBOEP**	**ΣAlkyl-mOPEs**	**BCEP**	**BCIPP**	**BDCIPP**	**ΣCl-mOPEs**	**ΣmOPEs**
**All volunteers (n=60)**												
[a] GM	72.8	1.36	102	460	17.5	124	963	3.39	16.44	3.80	44.5	1590
Max	4000	240	4000	6290	568	1750	6950	7550	1250	2090	7560	14000
75th	323	0.84	345	1020	114	418	1450	<LOQ	<LOQ	<LOQ	221	2420
Median	143	<LOQ	166	675	48	211	1100	<LOQ	<LOQ	<LOQ	13.7	1450
25th	19	<LOQ	32	387	1.61	23.9	613	<LOQ	<LOQ	<LOQ	13.7	1010
Min	<LOQ	<LOQ	1.69	<LOQ	<LOQ	<LOQ	119	<LOQ	<LOQ	<LOQ	13.7	279
[b] DF (%)	78.3	25.0		91.7	75	78.3		15.0	11.7	11.7		
[c] SD	846	51.7	843	1100	132	330	1270	1310	231	295	1370	2430
**Male (*n* = 36)**												
GM	42.2	0.88	54.1	432	17.3	97.8	854	3.58	15.9	3.42	41.7	1230
Max	2360	172	2360	4270	568	1750	6080	7550	787	2090	7560	14000
75th	181	<LOQ	226	1010	105	351	1330	<LOQ	<LOQ	<LOQ	102	1580
Median	133	<LOQ	138	627	41.7	127	957	<LOQ	<LOQ	<LOQ	13.7	1140
25th	<LOQ	<LOQ	14.6	270	3.63	<LOQ	561	<LOQ	<LOQ	<LOQ	13.7	800
Min	<LOQ	<LOQ	1.69	<LOQ	<LOQ	<LOQ	119	<LOQ	<LOQ	<LOQ	13.7	279
DF (%)	72.2	16.7		94.4	77.8	75.0		16.7	11.1	8.33		
**Female (*n* = 24)**												
GM	165	2.64	266	505	17.9	177	1150	3.12	17.3	4.46	49.2	2330
Max	4000	240	4000	6290	515	714	6950	6200	1250	923	6220	8410
75th	1060	33.5	1120	1090	134	470	1520	<LOQ	<LOQ	<LOQ	288	4900
Median	289	<LOQ	323	837	73.9	282	1130	<LOQ	<LOQ	<LOQ	13.7	2110
25th	31.6	<LOQ	157	548	<LOQ	92.2	989	<LOQ	<LOQ	<LOQ	13.7	1400
Min	<LOQ	<LOQ	1.7	<LOQ	<LOQ	<LOQ	192	<LOQ	<LOQ	<LOQ	13.7	359
DF (%)	87.5	37.5		87.5	70.8	95.8		12.5	12.5	16.7		

[a] GM: geometric mean; [b] DF: detection frequency; [c] SD: standard deviation.

Table S6. SG-adjusted geometric mean or median concentrations of urinary mOPEs worldwide (pg/mL).

Site	Year	*n*	Population	DPHP	BBOEP	BCEP	BCIPP	BDCIPP	Ref
South China	2015	411	children	280	50	1040	150	50	[52]
Norway	2012	244	mothers	630	nd			80	[53]
Norway	2012	112	children	1000	nd			230	[53]
		42	mothers		nd			<LOQ	[54]
		42	children	37000	<LOQ				[54]
USA	2005-2015	201	men	570			nd	460	[55]
USA	2005-2015	211	women	750			nd	690	[56]
USA	2010-2013	200	women	820				650	[57]
USA	2002-2005	349	pregnant	1310			6100	1850	[58]
	2005-2015	220	men	700				610	[59]
	2014-2016	203	children	50900			31900	80700	[60]
USA	1999-2000	310	pregnant	930			nd	410	[61]
USA	2001-2006	349	pregnant	1300			700	1900	[62]
USA	2010-2011	135	adults	170-142000					[63]
USA	2014	58	pregnant	930		310		1180	[64]
USA	2012-2014	41	children	2710				5470	[65]
USA	2015	28	mothers	1200			nd	2800	[66]
USA	2015	33	children	2500			nd	7400	[66]
USA	2012	11	female	8710				7600	[67]
USA	2015	40	adults	1160			nd	2060	[68]
USA	2014-2015	43	children	26500			7500	514000	[69]
USA	2012	53	adults	9090				4460	[70]
USA	2013-2014	19	mothers	68700			640	11000	[71]
USA	2013-2014	23	children	140000			460	251000	[71]
USA	2002-2007	45	men	270				120	[72]

Table S7. Correlation coefficient of mOPEs in human urine and OPEs in indoor dust.

	TEP	TBOEP	TCEP	TCIPP	TDCIPP	TPHP	TMPP
DPHP	-0.094	-0.101	-0.110	-0.073	-0.028	-0.228	-0.149

DMPP	-0.089	0.015	-0.082	-0.032	0.001	-0.114	-0.024
DEP	0.229	-0.291	0.001	-0.104	0.068	-0.119	-0.016
BEHP	-0.057	0.112	0.052	0.193	-0.018	-0.036	0.051
BBOEP	-0.104	-0.117	0.092	0.029	0.083	-0.133	-0.070
BCEP	0.083	-0.220	-0.002	-0.017	0.125	0.123	0.191
BCIPP	0.054	0.128	-0.019	0.075	0.170	0.284	0.182
BDCIPP	0.091*	0.131	.0450*	0.339*	0.049	0.007	0.224*

*. Correlation is significant at the 0.05 level (2-tailed). **. Correlation is significant at the 0.01 level (2-tailed).

Table S8. Correlation coefficients of mOPEs in human urine and OPEs in indoor atmospheric $PM_{2.5}$ samples.

	TEP	TBOEP	TCEP	TCIPP	TDCIPP	TPHP	TMPP
DPHP	-0.110	-0.301	-0.178	-0.128	-0.229	-0.328	-0.220
DMPP	-0.127	0.114	0.249	0.095	-0.304	-0.118	-0.151
DEP	0.365	0.167	-0.123	-0.161	0.071	-0.142	0.108
DEHP	-0.071	-0.095	-0.156	-0.262	-0.042	-0.180	0.008
BBOEP	0.292	0.078	0.250	0.094	-0.189	-0.108	-0.057
BCEP	0.355	-0.278	-0.175	-0.086	0.055	-0.203	0.075
BCIPP	-0.090	0.089	-0.201	-0.179	0.180	-0.189	-0.133
BDCIPP	-0.006	0.019	-0.163	-0.076	-0.057	-0.310	-0.091

*. Correlation is significant at the 0.05 level (2-tailed). **. Correlation is significant at the 0.01 level (2-tailed).

Table S9. The parameters of input files in PMF [73, 74].

	Symbol	Description
Con.	C_{ij}	Value of nonnegative; i for sample and j for species
Unc.	Uij	When $C_{ij} \leq$ MDL, Unc. = 5/6 MDL; When C_{ij} > MDL, Unc.=$[(\text{Error Fraction}\times\text{concentration})^2 + (\text{MDL})^2]^{1/2}$
Method Detection limit	MDL	3 $\text{mean}_{\text{blank}}$
Signal to noise ratio	S/N	Classification of species: it is categorized as "bad" if S/N<0.2; if 0.2<S/N<1, "weak"; if S/N>0.2, "strong"
Error Fraction	Error Fraction	20%

Table S10. Exposure parameters for children and adults [4].

Parameters	Unit	Abbr.	Value: Mean (95th percentile)	
			Children (1–11 year)	Adult (18–70 year)
Inhalation Rates	m^3/day	$R_{Inhalation}$	9.1 (13.3)	15.7 (21.3)
Time indoors	min/day	T_{indoor}	974 (1342)	948 (1428)
Body Weight	kg	BW	18.9	80
Skin surface area	m^2	SA	0.75 (0.94)	1.95 (2.36)
Dust Ingestion Rates	mg/day	$R_{Ingestion}$	60	30
Dust Adherence	g/cm^2	DA	0.096	0.096
Adsorption Factor	%	ADF	0.17	0.17

Table S11. Parameters used for calculation of total EDI and HI of OPEs in China [4,6,12].

	TPHP	TBOEP	TCEP	TCIPP	TDCIPP
RfDs (ng/kg bw/day)	70,000	15,000	7000	10,000	20,000
MWp (g/mol)	326	398	285	328	430
MWm (g/mol)	250	298	223	251	320
Fue (HLM)	0.09	0.81	0.07	0.33	0.46
Fue (S9)	0.04	0.16	0.13	0.28	0.68

References

1. Contini, D.; Cesari, D.; Conte, M.; Donateo, A., Application of PMF and CMB receptor models for the evaluation of the contribution of a large coal-fired power plant to PM concentrations. *Sci Total Environ* **2016,** 560-561, 131-140, doi: 10.1016/j.scitotenv.2016.04.031.
2. Wang, Y. J.; Liao, R. Q.; Liu, W. L.; Kannan, K.; Ohura, T.; Wu, M. H.; Ma, J., Chlorinated polycyclic aromatic hydrocarbons in surface sediment from Maowei Sea, Guangxi, China: occurrence, distribution, and source apportionment. *Environ Sci Pollut R* **2017,** 24, (19), 16241-16252, doi: 10.1007/s11356-017-9193-0.
3. Gao, B.; Guo, H.; Wang, X. M.; Zhao, X. Y.; Ling, Z. H.; Zhang, Z.; Liu, T. Y., Tracer-based source apportionment of polycyclic aromatic hydrocarbons in $PM_{2.5}$ in Guangzhou, southern China, using positive matrix factorization (PMF). *Environmental science and pollution research international* **2013,** 20, (4), 2398-409, doi: 10.1007/s11356-012-1129-0.
4. USEPA, Exposure Factors Handbook: 2011 Edition. *U.S. Environmental Protection Agency, Washington, DC, USA* **2011**, EPA/600/R-09/052F, http://www.epa.gov/ncea/efh.
5. Ali, N.; Shahzad, K.; Rashid, M. I.; Shen, H. Q.; Ismail, I. M. I.; Eqani, S. A. M. A. S., Currently used organophosphate and brominated flame retardants in the environment of China and other developing countries (2000-2016). *Environ Sci Pollut R* **2017,** 24, (23), 18721-18741, doi: 10.1007/s11356-017-9336-3.
6. USEPA, EPA Handbook: Update for Chapter 5 of the Exposure Factors Handbook: Soil and Dust Ingestion. **2017**, EPA/600/R-17/384F, https://cfpub.epa.gov/ncea/risk/recordisplay.cfm?deid=337521.
7. Ding, J. J.; Shen, X. L.; Liu, W. P.; Covaci, A.; Yang, F. X., Occurrence and risk assessment of organophosphate esters in drinking water from Eastern China. *Sci Total Environ* **2015,** 538, 959-965, doi: 10.1016/j.scitotenv.2015.08.101.
8. Li, J. F.; Zhang, Z. Z.; Ma, L. Y.; Zhang, Y.; Niu, Z. G., Implementation of USEPA RfD and SFO for improved risk assessment of organophosphate esters (organophosphate flame retardants and plasticizers). *Environ Int* **2018,** 114, 21-26, doi: 10.1016/j.envint.2018.02.027.
9. Hoffman, K.; Butt, C. M.; Webster, T. F.; Preston, E. V.; Hammel, S. C.; Makey, C.; Lorenzo, A. M.; Cooper, E. M.; Carignan, C.; Meeker, J. D.; Hauser, R.; Soubry, A.; Murphy, S. K.; Price, T. M.; Hoyo, C.; Mendelsohn, E.; Congleton, J.; Daniels, J. L.; Stapleton, H. M., Temporal Trends in Exposure to Organophosphate Flame Retardants in the United States. *Environ Sci Tech Let* **2017,** 4, (3), 112-118, doi: 10.1021/acs.estlett.6b00475.
10. Li, J. F.; Wang, C.; Du, L.; Lv, Z. W.; Li, X. N.; Hu, X. P.; Niu, Z. G.; Zhang, Y., Did municipal solid waste landfill have obvious influence on polychlorinated dibenzo-p-dioxins and polychlorinated dibenzofurans (PCDD/Fs) in ambient air: A case study in East China. *Waste Manage* **2017,** 62, 169-176, doi: 10.1016/j.wasman.2017.02.014.
11. Hoffman, K.; Gearhart-Serna, L.; Lorber, M.; Webster, T. F.; Stapleton, H. M., Estimated Tris(1,3-dichloro-2-propyl) Phosphate Exposure Levels for US Infants Suggest Potential Health Risks. *Environ Sci Tech Let* **2017,** 4, (8), 334-338, doi: 10.1021/acs.estlett.7b00196.
12. Van den Eede, N.; Maho, W.; Erratico, C.; Neels, H.; Covaci, A., First insights in the metabolism of phosphate flame retardants and plasticizers using human liver fractions. *Toxicol Lett* **2013,** 223, (1), 9-15, doi: 10.1016/j.toxlet.2013.08.012.
13. ICRP, ICRP publication 89: basic anatomical and physiological data for use in radiological protection: ref. *Ann. ICRP* **2002,** 32, 1-277.
14. Qiongpu Hu, L. X., Xiangying Zeng, Zhiqiang Yu, Co-occurrence and distribution of organophosphate tri- and di-esters in indoor dust from different indoor environments in Guangzhou and their potential human health risk. *Environ Pollut* **2020,** 262, 1 1431 1, doi: 10.1016/j.envpol.2020.114311.
15. Wang, Y.; Yao, Y. M.; Han, X. X.; Li, W. H.; Zhu, H. K.; Wang, L.; Sun, H. W.; Kannan, K., Organophosphate di- and tri-esters in indoor and outdoor dust from China and its implications for human exposure. *Sci Total Environ* **2020,** 700, doi: 10.1016/j.scitotenv.2019.134502.
16. Tan, H. L.; Yang, L.; Yu, Y. J.; Guan, Q. X.; Liu, X. T.; Li, L. Z.; Chen, D., Co-Existence of Organophosphate Di- and Tri-Esters in House Dust from South China and Midwestern United States: Implications for Human Exposure. *Environ Sci Technol* **2019,** 53, (9), 4784-4793, doi: 10.1021/acs.est.9b00229.
17. He, C. T.; Zheng, J.; Qiao, L.; Chen, S. J.; Yang, J. Z.; Yuan, J. G.; Yang, Z. Y.; Mai, B. X., Occurrence of organophosphorus flame retardants in indoor dust in multiple microenvironments of southern China and implications for human exposure. *Chemosphere* **2015,** 133, 47-52, doi: 10.1016/j.chemosphere.2015.03.043.
18. Zeng, X. Y.; Wu, Y.; Liu, Z. Y.; Gao, S. T.; Yu, Z. Q., Occurrence and Distribution of Organophosphate Ester Flame Retardants in Indoor Dust and Their Potential Health Exposure Risk. *Environ Toxicol Chem* **2018,** 37, (2), 345-352, doi: 10.1002/etc.3996.

19. Wu, M.; Yu, G.; Cao, Z. G.; Wu, D. K.; Liu, K.; Deng, S. B.; Huang, J.; Wang, B.; Wang, Y. J., Characterization and human exposure assessment of organophosphate flame retardants in indoor dust from several microenvironments of Beijing, China. *Chemosphere* **2016,** 150, 465-471, doi: 10.1016/j.chemosphere.2015.12.111.
20. Peng, C. F.; Tan, H. L.; Guo, Y.; Wu, Y.; Chen, D., Emerging and legacy flame retardants in indoor dust from East China. *Chemosphere* **2017,** 186, 635-643, doi: 10.1016/j.chemosphere.2017.08.038.
21. Tan, H. L.; Peng, C. F.; Guo, Y.; Wang, X. D.; Wu, Y.; Chen, D., Organophosphate Flame Retardants in House Dust from South China and Related Human Exposure Risks. *B Environ Contam Tox* **2017,** 99, (3), 344-349, doi: 10.1007/s00128-017-2120-8.
22. He, R. W.; Li, Y. Z.; Xiang, P.; Li, C.; Zhou, C. Y.; Zhang, S. J.; Cui, X. Y.; Ma, L. Q., Organophosphorus flame retardants and phthalate esters in indoor dust from different microenvironments: Bioaccessibility and risk assessment. *Chemosphere* **2016,** 150, 528-535, doi: 10.1016/j.chemosphere.2015.10.087.
23. Yadav, I. C.; Devi, N. L.; Zhong, G. C.; Li, J.; Zhang, G.; Covaci, A., Occurrence and fate of organophosphate ester flame retardants and plasticizers in indoor air and dust of Nepal: Implication for human exposure. *Environ Pollut* **2017,** 229, 668-678, doi: 10.1016/j.envpol.2017.06.089.
24. Kim, J. W.; Isobe, T.; Sudaryanto, A.; Malarvannan, G.; Chang, K. H.; Muto, M.; Prudente, M.; Tanabe, S., Organophosphorus flame retardants in house dust from the Philippines: occurrence and assessment of human exposure. *Environ Sci Pollut R* **2013,** 20, (2), 812-822, doi: 10.1007/s11356-012-1237-x.
25. Ali, N.; Eqani, S. A. M. A. S.; Ismail, I. M. I.; Malarvannan, G.; Kadi, M. W.; Albar, H. M. S.; Rehan, M.; Covaci, A., Brominated and organophosphate flame retardants in indoor dust of Jeddah, Kingdom of Saudi Arabia: Implications for human exposure. *Sci Total Environ* **2016,** 569, 269-277, doi: 10.1016/j.scitotenv.2016.06.093.
26. Ali, N.; Ali, L.; Mehdi, T.; Dirtu, A. C.; Al-Shammari, F.; Neels, H.; Covaci, A., Levels and profiles of organochlorines and flame retardants in car and house dust from Kuwait and Pakistan: implication for human exposure via dust ingestion. *Environ Int* **2013,** 55, 62-70, doi: 10.1016/j.envint.2013.02.001.
27. Mizouchi, S.; Ichiba, M.; Takigami, H.; Kajiwara, N.; Takamuku, T.; Miyajima, T.; Kodama, H.; Someya, T.; Ueno, D., Exposure assessment of organophosphorus and organobromine flame retardants via indoor dust from elementary schools and domestic houses. *Chemosphere* **2015,** 123, 17-25, doi: 10.1016/j.chemosphere.2014.11.028.
28. Abdallah, M. A.; Covaci, A., Organophosphate Flame Retardants in Indoor Dust from Egypt: Implications for Human Exposure. *Environ Sci Technol* **2014,** 48, (9), 4782-4789, doi: 10.1021/es501078s.
29. Ali, N.; Dirtu, A. C.; Van den Eede, N.; Goosey, E.; Harrad, S.; Neels, H.; t Mannetje, A.; Coakley, J.; Douwes, J.; Covaci, A., Occurrence of alternative flame retardants in indoor dust from New Zealand: indoor sources and human exposure assessment. *Chemosphere* **2012,** 88, (11), 1276-82, doi: 10.1016/j.chemosphere.2012.03.100.
30. He, C.; Wang, X. Y.; Thai, P.; Mueller, J. F.; Gallen, C.; Li, Y.; Baduel, C., Development and validation of a multi-residue method for the analysis of brominated and organophosphate flame retardants in indoor dust. *Talanta* **2017,** 164, 503-510, doi: 10.1016/j.talanta.2016.10.108.
31. Bergh, C.; Torgrip, R.; Emenius, G.; Ostman, C., Organophosphate and phthalate esters in air and settled dust - a multi-location indoor study. *Indoor Air* **2011,** 21, (1), 67-76, doi: 10.1111/j.1600-0668.2010.00684.x.
32. Luongo, G.; Ostman, C., Organophosphate and phthalate esters in settled dust from apartment buildings in Stockholm. *Indoor Air* **2016,** 26, (3), 414-425, doi: 10.1111/ina.12217.
33. van den Eede, N.; Dirtu, A. C.; Neels, H.; Covaci, A., Analytical developments and preliminary assessment of human exposure to organophosphate flame retardants from indoor dust. *Environ Int* **2011,** 37, (2), 454-461, doi: 10.1016/j.envint.2010.11.010.
34. Cristale, J.; Hurtado, A.; Gomez-Canela, C.; Lacorte, S., Occurrence and sources of brominated and organophosphorus flame retardants in dust from different indoor environments in Barcelona, Spain. *Environ Res* **2016,** 149, 66-76, doi: 10.1016/j.envres.2016.05.001.
35. Brommer, S.; Harrad, S.; Van den Eede, N.; Covaci, A., Concentrations of organophosphate esters and brominated flame retardants in German indoor dust samples. *Journal of environmental monitoring : JEM* **2012,** 14, (9), 2482-7, doi: 10.1039/c2em30303e.
36. Brommer, S.; Harrad, S., Sources and human exposure implications of concentrations of organophosphate flame retardants in dust from UK cars, classrooms, living rooms, and offices. *Environ Int* **2015,** 83, 202-7, doi: 10.1016/j.envint.2015.07.002.
37. Brandsma, S. H.; de Boer, J.; van Velzen, M. J.; Leonards, P. E., Organophosphorus flame retardants (PFRs) and plasticizers in house and car dust and the influence of electronic equipment. *Chemosphere* **2014**, doi: 10.1016/j.chemosphere.2014.02.036.
38. Zhou, L.; Hiltscher, M.; Puttmann, W., Occurrence and human exposure assessment of organophosphate flame retardants in indoor dust from various microenvironments of the Rhine/Main region, Germany. *Indoor Air* **2017,** 27, (6), 1113-1127, doi: 10.1111/ina.12397.
39. Schreder, E. D.; La Guardia, M. J., Flame Retardant Transfers from U.S. Households (Dust and Laundry Wastewater) to the Aquatic Environment. *Environ Sci Technol* **2014,** 48, (19), 11575-11583, doi: 10.1021/es502227h.
40. Stapleton, H. M.; Klosterhaus, S.; Eagle, S.; Fuh, J.; Meeker, J. D.; Blum, A.; Webster, T. F., Detection of Organophosphate Flame Retardants in Furniture Foam and US House Dust. *Environ Sci Technol* **2009,** 43, (19), 7490-7495, doi: 10.1021/es9014019.
41. Dirtu, A. C.; Ali, N.; Van den Eede, N.; Neels, H.; Covaci, A., Country specific comparison for profile of chlorinated, brominated and phosphate organic contaminants in indoor dust. Case study for Eastern Romania, 2010. *Environ Int* **2012,** 49, 1-8, doi: 10.1016/j.envint.2012.08.002.
42. Cristale, J.; Bele, T. G. A.; Lacorte, S.; de Marchi, M. R. R., Occurrence and human exposure to brominated and organophosphorus flame retardants via indoor dust in a Brazilian city. *Environ Pollut* **2018,** 237, 695-703, doi: 10.1016/j.envpol.2017.10.110.
43. Li, H. L.; Liu, L. Y.; Zhang, Z. F.; Ma, W. L.; Sverko, E.; Zhang, Z.; Song, W. W.; Sun, Y.; Li, Y. F., Semi-volatile organic com-

pounds in infant homes: Levels, influence factors, partitioning, and implications for human exposure. *Environ Pollut* **2019,** 251, 609-618, doi: 10.1016/j.envpol.2019.05.048.
44. Tokumura, M.; Hatayama, R.; Tatsu, K.; Naito, T.; Takeda, T.; Raknuzzaman, M.; Habibullah-Al-Mamun, M.; Masunaga, S., Organophosphate flame retardants in the indoor air and dust in cars in Japan. *Environ Monit Assess* **2017,** 189, (2) , doi: 10.1007/s10661-016-5725-1.
45. Yadav, I. C.; Devi, N. L.; Kumar, A.; Li, J.; Zhang, G., Airborne brominated, chlorinated and organophosphate ester flame retardants inside the buildings of the Indian state of Bihar: Exploration of tag source and human exposure. *Ecotox Environ Safe* **2020,** 191, doi: 10.1016/j.ecoenv.2020.110212.
46. Kim, U. J.; Wang, Y.; Li, W. H.; Kannan, K., Occurrence of and human exposure to organophosphate flame retardants/plasticizers in indoor air and dust from various microenvironments in the United States. *Environ Int* **2019,** 125, 342-349, doi: 10.1016/j.envint.2019.01.065.
47. Liu, X. T.; Chen, D.; Yu, Y. J.; Zeng, X. W.; Li, L. Z.; Xie, Q. T.; Yang, M.; Wu, Q. Z.; Dong, G. H., Novel Organophosphate Esters in Airborne Particulate Matters: Occurrences, Precursors, and Selected Transformation Products (vol 54, pg 13771, 2020). *Environ Sci Technol* **2021,** 55, (3), 2170-2170, doi: 10.1021/acs.est.1c00105.
48. Wong, F.; de Wit, C. A.; Newton, S. R., Concentrations and variability of organophosphate esters, halogenated flame retardants, and polybrominated diphenyl ethers in indoor and outdoor air in Stockholm, Sweden. *Environ Pollut* **2018,** 240, 514-522, doi: 10.1016/j.envpol.2018.04.086.
49. Tran, L. K.; He, C.; Phuc, D. H.; Toms, L. M. L.; Wang, X. Y.; Xiu, M.; Mueller, J. F.; Covaci, A.; Morawska, L.; Thai, P. K., Monitoring the levels of brominated and organophosphate flame retardants in passenger cars: Utilisation of car air filters as active samplers. *Journal Of Environmental Sciences* **2020,** 91, 142-150, doi: 10.1016/j.jes.2020.01.014.
50. Hartmann, P. C.; Burgi, D.; Giger, W., Organophosphate flame retardants and plasticizers in indoor air. *Chemosphere* **2004,** 57, (8), 781-787, doi: 10.1016/j.chemosphere.2004.08.051.
51. Sakhi, A. K.; Cequier, E.; Becher, R.; Bolling, A. K.; Borgen, A. R.; Schlabach, M.; Schmidbauer, N.; Becher, G.; Schwarze, P.; Thomsena, C., Concentrations of selected chemicals in indoor air from Norwegian homes and schools. *Sci Total Environ* **2019,** 674, 1-8, doi: 10.1016/j.scitotenv.2019.04.086.
52. Chen, Y.; Fang, J. Z.; Ren, L.; Fan, R. F.; Zhang, J. Q.; Liu, G. H.; Zhou, L.; Chen, D. Y.; Yu, Y. X.; Lu, S. Y., Urinary metabolites of organophosphate esters in children in South China: Concentrations, profiles and estimated daily intake. *Environ Pollut* **2018,** 235, 358-364, doi: 10.1016/j.envpol.2017.12.092.
53. Cequier, E.; Sakhi, A. K.; Marce, R. M.; Becher, G.; Thomsen, C., Human exposure pathways to organophosphate triesters - A biomonitoring study of mother-child pairs. *Environ Int* **2015,** 75, 159-165, doi: 10.1016/j.envint.2014.11.009.
54. Cequier, E.; Marce, R. M.; Becher, G.; Thomsen, C., A high-throughput method for determination of metabolites of organophosphate flame retardants in urine by ultra performance liquid chromatography-high resolution mass spectrometry. *Anal Chim Acta* **2014,** 845, 98-104, doi: 10.1016/j.aca.2014.06.026.
55. Carignan, C. C.; Minguez-Alarcon, L.; Williams, P. L.; Meeker, J. D.; Stapleton, H. M.; Butt, C. M.; Toth, T. L.; Ford, J. B.; Hauser, R.; Team, E. S., Paternal urinary concentrations of organophosphate flame retardant metabolites, fertility measures, and pregnancy outcomes among couples undergoing in vitro fertilization. *Environ Int* **2018,** 111, 232-238, doi: 10.1016/j.envint.2017.12.005.
56. Carignan, C. C.; Minguez-Alarcon, L.; Butt, C. M.; Williams, P. L.; Meeker, J. D.; Stapleton, H. M.; Toth, T. L.; Ford, J. B.; Hauser, R.; Team, E. S., Urinary Concentrations of Organophosphate Flame Retardant Metabolites and Pregnancy Outcomes among Women Undergoing in Vitro Fertilization. *Environ Health Persp* **2017,** 125, (8) , doi: 10.1289/EHP1021.
57. Deziel, N. C.; Yi, H. D.; Stapleton, H. M.; Huang, H.; Zhao, N.; Zhang, Y. W., A case-control study of exposure to organophosphate flame retardants and risk of thyroid cancer in women. *Bmc Cancer* **2018,** 18, doi: 10.1186/s12885-018-4553-9.
58. Hoffman, K.; Stapleton, H. M.; Lorenzo, A.; Butt, C. M.; Adair, L.; Herring, A. H.; Daniels, J. L., Prenatal exposure to organophosphates and associations with birthweight and gestational length. *Environ Int* **2018,** 116, 248-254, doi: 10.1016/j.envint.2018.04.016.
59. Ingle, M. E.; Minguez-Alarcon, L.; Carignan, C. C.; Butt, C. M.; Stapleton, H. M.; Williams, P. L.; Ford, J. B.; Hauser, R.; Meeker, J. D.; Team, E. S., The association between urinary concentrations of phosphorous-containing flame retardant metabolites and semen parameters among men from a Check tor updates fertility clinic. *Int J Hyg Envir Heal* **2018,** 221, (5), 809-815, doi: 10.1016/j.ijheh.2018.05.001.
60. Phillips, A. L.; Hammel, S. C.; Hoffman, K.; Lorenzo, A. M.; Chen, A.; Webster, T. F.; Stapleton, H. M., Children's residential exposure to organophosphate ester flame retardants and plasticizers: Investigating exposure pathways in the TESIE study. *Environ Int* **2018,** 116, 176-185, doi: 10.1016/j.envint.2018.04.013.
61. Castorina, R.; Butt, C.; Stapleton, H. M.; Avery, D.; Harley, K. G.; Holland, N.; Eskenazi, B.; Bradman, A., Flame retardants and their metabolites in the homes and urine of pregnant women residing in California (the CHAMACOS cohort). *Chemosphere* **2017,** 179, 159-166, doi: 10.1016/j.chemosphere.2017.03.076.
62. Hoffman, K.; Lorenzo, A.; Butt, C. M.; Adair, L.; Herring, A. H.; Stapleton, H. M.; Daniels, J. L., Predictors of urinary flame retardant concentration among pregnant women. *Environ Int* **2017,** 98, 96-101, doi: 10.1016/j.envint.2016.10.007.
63. Preston, E. V.; McClean, M. D.; Henn, B. C.; Stapleton, H. M.; Braverman, L. E.; Pearce, E. N.; Makey, C. M.; Webster, T. F., Associations between urinary diphenyl phosphate and thyroid function. *Environ Int* **2017,** 101, 158-164, doi: 10.1016/j.envint.2017.01.020.
64. Romano, M. E.; Hawley, N. L.; Eliot, M.; Calafat, A. M.; Jayatilaka, N. K.; Kelsey, K.; McGarvey, S.; Phipps, M. G.; Savitz, D. A.; Werner, E. F.; Braun, J. M., Variability and predictors of urinary concentrations of organophosphate flame retardant metabo-

lites among pregnant women in Rhode Island. *Environ Health-Glob* **2017,** 16, doi: 10.1186/s12940-017-0247-z.
65. Thomas, M. B.; Stapleton, H. M.; Dills, R. L.; Violette, H. D.; Christakis, D. A.; Sathyanarayana, S., Demographic and dietary risk factors in relation to urinary metabolites of organophosphate flame retardants in toddlers. *Chemosphere* **2017,** 185, 918-925, doi: 10.1016/j.chemosphere.2017.07.015.
66. Butt, C. M.; Hoffman, K.; Chen, A.; Lorenzo, A.; Congleton, J.; Stapleton, H. M., Regional comparison of organophosphate flame retardant (PFR) urinary metabolites and tetrabromobenzoic acid (TBBA) in mother-toddler pairs from California and New Jersey. *Environ Int* **2016,** 94, 627-634, doi: 10.1016/j.envint.2016.06.029.
67. Carignan, C. C.; Fang, M. L.; Stapleton, H. M.; Heiger-Bernays, W.; McClean, M. D.; Webster, T. F., Urinary biomarkers of flame retardant exposure among collegiate US gymnasts. *Environ Int* **2016,** 94, 362-368, doi: 10.1016/j.envint.2016.06.030.
68. Hammel, S. C.; Hoffman, K.; Webster, T. F.; Anderson, K. A.; Stapleton, H. M., Measuring Personal Exposure to Organophosphate Flame Retardants Using Silicone Wristbands and Hand Wipes. *Environ Sci Technol* **2016,** 50, (8), 4483-4491, doi: 10.1021/acs.est.6b00030.
69. Hoffman, K.; Butt, C. M.; Chen, A.; Limkakeng, A. T.; Stapleton, H. M., High Exposure to Organophosphate Flame Retardants in Infants: Associations with Baby Products. *Environ Sci Technol* **2015,** 49, (24), 14554-14559, doi: 10.1021/acs.est.5b03577.
70. Hoffman, K.; Garantziotis, S.; Birnbaum, L. S.; Stapleton, H. M., Monitoring Indoor Exposure to Organophosphate Flame Retardants: Hand Wipes and House Dust. *Environ Health Persp* **2015,** 123, (2), 160-165, doi: 10.1289/ehp.1408669.
71. Butt, C. M.; Congleton, J.; Hoffman, K.; Fang, M. L.; Stapleton, H. M., Metabolites of Organophosphate Flame Retardants and 2-Ethylhexyl Tetrabromobenzoate in Urine from Paired Mothers and Toddlers. *Environ Sci Technol* **2014,** 48, (17), 10432-10438, doi: 10.1021/es5025299.
72. Meeker, J. D.; Cooper, E. M.; Stapleton, H. M.; Hauser, R., Urinary Metabolites of Organophosphate Flame Retardants: Temporal Variability and Correlations with House Dust Concentrations. *Environ Health Persp* **2013,** 121, (5), 580-585, doi: 10.1289/ehp.1205907.
73. Wang, Y. B.; Liu, C. W.; Kao, Y. H.; Jang, C. S., Characterization and risk assessment of PAH-contaminated river sediment by using advanced multivariate methods. *Sci Total Environ* **2015,** 524-525, 63-73, doi: 10.1016/j.scitotenv.2015.04.019.
74. Burger, J.; Fossi, C.; McClellan-Green, P.; Orlando, E. F., Methodologies, bioindicators, and biomarkers for assessing gender-related differences in wildlife exposed to environmental chemicals. *Environ Res* **2007,** 104, (1), 135-152, doi: 10.1016/j.envres.2006.08.002.
